# “Wait, Do I Need More Fiber?” Exploring UK Consumers’ Dietary Fiber-Related Awareness and White Bread as a Viable Solution to Promote Subsequent Intake

**DOI:** 10.1016/j.cdnut.2024.104430

**Published:** 2024-07-26

**Authors:** Victoria Norton, Carol Wagstaff, Julia Rodriguez Garcia, Alison Lovegrove, Peter Shewry, Mark Charlton, Nicola Gillett, Marcus John Tindall, Stella Lignou

**Affiliations:** 1 Department of Food and Nutritional Sciences, Harry Nursten, University of Reading, Reading, United Kingdom; 2 Nutrition and Food Science Area, Preventive Medicine and Public Health, Food Science, Toxicology and Forensic Medicine Department, Faculty of Pharmacy, Universitat de València, Avda. Vicent Andrés Estellés, s/n, 46100 Burjassot, València, Spain; 3 Sustainable Soils and Crops, Rothamsted Research, Harpenden, Hertfordshire, AL5 2JQ, United Kingdom; 4 Allied Technical Centre, 1 Vanwall Place, Vanwall Business Park, Maidenhead, Berkshire, SL6 4UF, United Kingdom; 5 Department of Mathematics and Statistics, University of Reading, Reading, United Kingdom; 6 Institute of Cardiovascular and Metabolic Research, University of Reading, Whiteknights, Reading, RG6 6AA, United Kingdom

**Keywords:** dietary fiber, focus groups, consumer engagement, staple foods, white bread

## Abstract

**Background:**

Sufficient dietary fiber consumption is associated with well-established health benefits, yet such intake is currently suboptimal globally. Thus, there is interest in developing strategies to improve dietary fiber intake. One such approach is to increase the dietary fiber content of staple foods, but this needs relevant investigation.

**Methods:**

Forty-two United Kingdom (UK) based consumers (18–76 y) were recruited to take part in seven focus group sessions investigating: (i) key factors in food choice; (ii) dietary fiber-related knowledge, awareness, consumption habits, and engagement levels; (iii) willingness to consume dietary fiber-rich staple foods; and (iv) gain initial feedback on dietary fiber-rich breads.

**Results:**

Overall, key dietary fiber themes emerged such as knowledge (benefits, foods, recommendations and labeling), consumption (not measuring intake), barriers (convenience and knowledge), resources (education and public appeal), and topics (food examples and cooking). Consumers were positive *per se* to the idea of dietary fiber-rich staple foods but with various caveats (no changes in appearance, taste, and cost). White bread trends were centered around context (sandwich and toast), habit (comfort food), preferences (soft and fresh), and consumption is variable (daily to less often). In addition, consumers’ preferred labeling strategy for dietary fiber-rich breads was predominately focused on transparency and visibility. Overall, the newly developed breads were well received demonstrating the potential of our prototypes to fit into the white bread market; however, additional consumer insights are needed.

**Conclusion:**

Our findings recommend combining education with a personalized element of advice, coupled with a collective effort from the government and food industry, as essential to help encourage a step-change in dietary fiber consumption in the UK population.

## Introduction

Dietary fiber is an essential dietary component and is associated with well-proven health benefits such as reduced cardiovascular disease, coronary events, stroke, type 2 diabetes, and cancer (colorectal) risk [1]. However, most of the UK population consume below the dietary fiber recommendation of 30 g/d; hence, increasing such intake could have noteworthy public health benefits [2]. It is likely that a number of factors are driving the poor uptake such as perceived cost, inadequate cooking skills, limited sensory appeal, side effects, lack of knowledge, and insufficient on-pack labeling [[3], [4], [5], [6], [7], [8], [9], [10], [11]]. More broadly, overriding food choice factors (such as societal, individual differences, and food aspects) have a fundamental role in purchasing decisions; thus, clarifying such factors could help to support food system transformation [12]. Therefore, there is a collective effort within the food industry to help overcome the widespread dietary fiber-related deficit via feasible, cost-effective, and readily consumed solutions.

Staple foods provide an ideal basis to help increase dietary fiber intake and bread fits within this remit as well as being commonly consumed globally and considered affordable [13]. In addition, bread is typically consumed 2–6 times a week, often as a sandwich or toast by the UK consumers [14]. More specifically, white bread (prepacked) is the market leader in terms of bread sales in the UK; hence, an ideal and popular bread type that could be used to support higher dietary fiber consumption rates [14,15]. However, white flour (and bread) is produced by milling the grain to remove the bran and germ which leads to nutrient losses and subsequently negatively impacts disease risk; therefore, enhancing this staple food source quality could have noteworthy public health implications [[16], [17], [18], [19]]. Hence, researchers have focused on developing novel wheat types (using conventional breeding strategies) with higher contents of the major dietary fiber component (arabinoxylan) in white flour [20]. Recently, such lines have been used to make white bread with relatively positive sensory and physical properties (e.g., smaller slice height, higher water activity/moisture content, and darker color) [21]. However, additional research is needed to understand consumers’ insights in relation to dietary fiber-rich white breads, so that such breads meet consumer expectations.

It is fundamental that appropriate methodologies are used to capture relevant consumer needs, attitudes, and perceptions; accordingly, qualitative approaches such as using focus groups enable group interaction via an individual/shared perspective as well as gaining in-depth insight into knowledge and experiences (including what, how, and why) on a particular topic [22,23]. In addition, focus groups are useful at an early stage of research to explore the topic and understand key issues before future quantitative investigation [23]. It is evident that a range of focus groups in different countries (e.g., Australia, Iran, Singapore, United States of America, and UK) have been successfully conducted predominately focused on promoting dietary fiber-rich foods (such as whole grains) [9,[24], [25], [26], [27], [28], [29], [30]]. However, such an approach would also be appropriate for higher dietary fiber white bread because this could be a potentially viable route to support increased dietary fiber consumption [31]. Currently, this area has received less attention, most likely because of the need to fortify foods with exogenous fiber, which can modulate cost and processing levels [31].

Accordingly, to address the associated research gaps, our study used focus groups as a medium to initiate conversation as well as to enable tastings of different white bread prototypes (varying in dietary fiber content) to understand initial consumer acceptability. This latter point is considered a limitation of previous dietary fiber focus group-related studies and could help overcome any potential food neophobia concerns (e.g., reluctance/avoidance to eat novel foods) [9,25,26,30,32]. In addition, the overall emphasis was on providing the consumers with the relevant background (such as what is dietary fiber and why it is important) so that they understood the need for easy strategies to incorporate dietary fiber into everyday life and subsequently promote engagement. Accordingly, our study aimed to (*1*) investigate consumers’ key factors in food choice; (*2*) explore consumers’ dietary fiber-related knowledge, awareness, consumption habits, and engagement levels; (*3*) understand consumers’ willingness to consume staple foods higher in dietary fiber; and (*4*) gain initial feedback on dietary fiber-rich white bread prototypes, in a UK context.

## Methods

### Study overview

Forty-two consumers (42.5 ± 17.7 y; range: 18–76 y; 31% male and 69% female) were recruited to take part in focus groups (between 75 and 90 min in length) in Reading either at the University or in community settings during September–November 2023. It was apparent that seven sessions (on average of six consumers per focus group) would be sufficient to reach data saturation [[33], [34], [35]]. Healthy consumers (aged ≥18 y, willing to discuss/share ideas, and with no allergies or intolerances) were recruited from the Reading area and/or attended the local community center regularly. Consumers had the study fully explained, provided informed consent, and were notified that the data would be pseudo-anonymized as well as their right to withdraw at any time. The study received a favorable opinion for conduct by the University of Reading School of Chemistry, Food and Pharmacy Research Ethics Committee (study number: 38/2023) as well as complying with the Declaration of Helsinki.

### Focus group design

**FIGURE 1:**
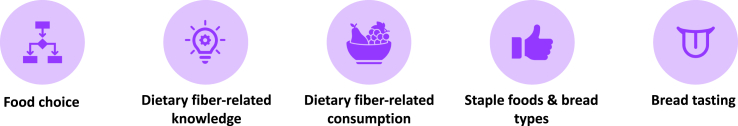
Summary of the key areas covered during the focus group sessions.

The sessions were centered around five key areas (as summarized in Figure 1) where a semi-structured discussion guide was used for all sessions. Input from our previous work [11] was used to inform the discussion guide. All sessions were conducted by the same moderator to enable consistency and audio recorded using Microsoft Teams (version 1.600.30658) so that sessions could be subsequently transcribed verbatim.

All focus groups started with an icebreaker task (e.g., what is your favorite hobby and food) to encourage conversation and participation. Consumers were informed how the session would work as well as having an emphasis on no right or wrong answers and contribution as they felt appropriate. In addition, consumers were asked about key factors in food choice to understand the main drivers as well as interest in their diet so as to capture initial engagement levels. The second section focused on understanding consumers’ dietary fiber-related knowledge and they were asked to describe: (ii) what do you know about dietary fiber (including benefits and food-based examples)?; (ii) what are the dietary fiber recommendations?; and (iii) do you check the dietary fiber content of foods? The third section explored consumers’ dietary fiber consumption habits and engagement levels where they discussed: (*1*) commonly consumed dietary fiber-rich foods; (*2*) barriers associated with dietary fiber; (*3*) current dietary fiber intake; and (*4*) potential dietary fiber resources and topics. The fourth section aimed to understand: (a) initial reactions for staple foods (e.g., rice, pasta, bread, etc.) higher in dietary fiber; (b) commonly consumed bread types; (c) views on white bread and consumption habits; and (d) expectations for dietary fiber-rich bread.

The final section focused on tasting three different white breads varying in dietary fiber content so as to gain qualitative feedback. The rationale for selecting the three breads (e.g., on-the-market control, Minax-100, and Minax-168) was based on sensory and physical properties results from our previous work [21]. In brief, the Minax lines (with a range of dietary fiber contents) were grown and milled as reported previously [20,21,36], whereas the on-the-market control used commercial wheat lines [21]. The breads were baked in accordance with the commercial bakers’ in-house procedures (800 g into a four-piece lidded loaf using a four-strap tin) using the Chorleywood breadmaking process and baked at 250°C for 24 min [21]. Consumers were presented (monadically in a balanced order across the seven sessions) with a slice of bread (40 g; Table 1) and asked to provide comments relating to the bread. In addition, they were asked to select their most preferred bread (post-initial evaluation) and purchase intentions as well as provide suggestions on how to improve the breads. To finish, consumers were asked to express their views on labeling (e.g., health by stealth compared with on-pack information), identify the bread they perceived to be higher in dietary fiber, and whether now they would modulate their dietary fiber intake.

**TABLE 1:** Overview of scanned bread slices (scans reduced to 45%)

Control	Minax-100	Minax-168
		

### Data analysis

The transcribed data was coded in NVivo (release 14.23.0) to identify, analyze, and report emerging themes (e.g., thematic analysis) using an inductive data-driven approach [37,38]. In brief, the data were analyzed in accordance with the Braun and Clarke step-by-step guide: (i) data familiarization; (ii) initial codes generation; (iii) themes development; (iv) reviewing themes; (v) defining/naming themes; and (vi) reporting, as well as adhering to the good practice process checklist (such as transcription, coding, analysis, overall, and report) for thematic analysis [38]. The codebook was subsequently cross-checked by a second author to ensure appropriate data representation as well as to enable a consensus on the coding and relevant themes (Supplemental Figure 1).

## Results

### Food choice

**FIGURE 2:**
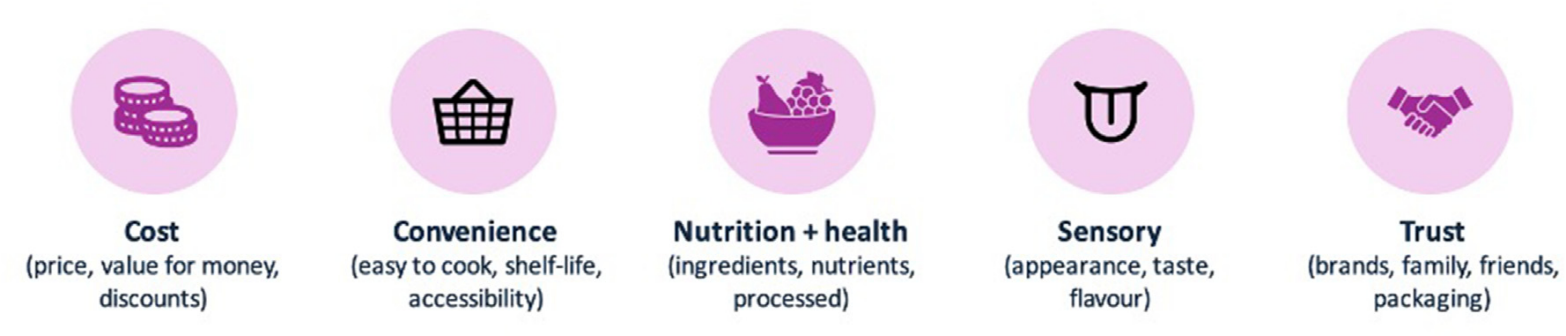
Summary of consumers’ key drivers in food choice.

Five main themes emerged relating to key factors in food choice: (*1*) cost was dominating the conversation such as *“value for money comes first – I am looking for the most amount for the least amount of money*” and *“price is always part of it*”; (*2*) convenience was also considered fundamental especially in terms of accessibility *“large supermarkets can be far away and not all have free delivery*” and easy to cook *“pasta and sauce – easy – fills you up*”; (*3*) nutritional and health aspects namely ingredients *“I packet flip as I am vegan, so I don’t get caught out*” and nutrients *“I like the traffic light system on the front-of-pack* *– green (healthy) vs red (unhealthy)*”; (*4*) sensory appeal covering appearance *“looks like*” and palatability *“taste, flavour*”; and *5*) trust resulted in an emphasis on the essentials *“focus on the basics – same brands*” and trusted brands/individuals *“happy to try new things if people explain it to me*” (Figure 2 and Table 2).

**TABLE 2:** Summary of additional quotes within corresponding themes

Theme	Quotes
Food choice	*“I try to balance everything the health, money, easy to cook and shelf-life*” F-23 *“towards the end of the month – you have £10 for three days – health may not come into it – itis just what can I eat for £10*” F-28
Dietary fiber knowledge and awareness	*“important for gut health but get a bit overwhelmed and confused with it all*” F-22 *“news to me that fiberdid anything for your heart just purely digestive*” F-21 *“I am not sure I did realise there are dietary fiberrecommendations in the UK*” M-70 *“if it is so good for you why is it not on the front?*” F-51
Dietary fiber consumption and engagement	*“I don’t know what really contains fiber*” F-35 *“if you have kids running around and shopping you don’t want to be there looking at the ingredients lists*” F-35 *“5-a-day is easy you can count on your fingers…bananas, peas, carrots, etc..*” M-59 *“information should be readily available not by accident*” F-40
Staple foods and bread types	*“if itis a price for everyone then that could work*” M-53 *“I would be wary as itis a change*” F-40 *“not dense - must keep softness!*” F-54 *“mine is white bread mainly as that was what I had growing up*” F-22 *“should I ignore the healthiness today and have white bread”* F-64 *“I would like it to be a natural process rather than it being injected*” M-30
Bread tasting	*“crust is tastier*” F-28 *“larger size – will it toast?*” F-70
Overall feedback	*“I didn’t realise some of the foods had fiber*” M-70 *“I would consider trying or having more fiber*” F-22

### Dietary fiber knowledge and awareness

**FIGURE 3:**
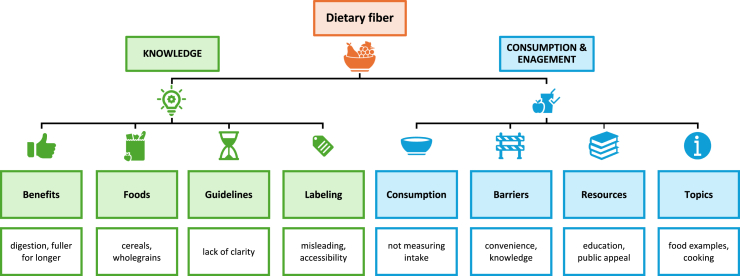
Overview of consumers’ key dietary fiber-related themes.

Overall, it was evident that dietary fiber is not at the forefront of consumers’ minds; therefore, contributing to the widespread confusion and poor awareness such as *“it is not good for you or is it*” and *“fiber is brown*.” More specifically, key themes relating to dietary fiber have been summarized in Figure 3 and Table 2. It was evident that there was a strong association between dietary fiber and digestive function *“guts happy, gut health, keep things moving*” as well as with satiety *“fuller for longer, weight management*.” However, in most cases, consumers were unaware that dietary fiber had additional health benefits such as reducing disease risk. Consumers cited *“cereals, whole grains, vegetables, pulses/beans and fruits*” as key sources of dietary fiber as well as the role of marketing in increasing subsequent awareness *“cereals are the ones that comes to mind – mainly from the marketing/packaging*.” There were also knowledge-related gaps *“what are good sources of fiber*” and *“what vegetables have fiber*?” Similarly, consumers’ awareness relating to the 30 g/d dietary fiber recommendations for the majority resulted in notable confusion *“I did not know the number*” and *“5-a-day – is this the same?*” This lack of clarity trend continued into the labeling discussion. For example, key themes related to poor accessibility *“I need my reading glasses to check back-of-pack, so I often do this at home*,” misleading on-pack information *“what is a portion size?*” and focus on fundamentals *“typically, not checking for fiber*.”

### Dietary fiber consumption and engagement

Dominant consumption and engagement themes are outlined in Figure 3 and Table 2. It was evident that consumers focused on eating by feeling *“I focus on feeling and listening to my body*” and not measuring intake *“I feel I get enough but I do not measure it and not sure what is absorbed at the same time*” with typical dietary fiber consumption patterns centered on fundamental, safe and familiar foods (e.g., baked beans, cereals, fruits, vegetables, and brown rice/pasta). In addition, there was an emphasis on lack of knowledge contributing to poor awareness *“it* *is not a topic widely discussed*” and *“I don’t know – I don’t look for it*” as well as the need for more support and information *“sell the benefit – what changes will you notice and what will it fix*” and *“taste before you buy in supermarkets – might help me*”. Consumers cited a number of noteworthy dietary fiber-related barriers including: (i) insufficient knowledge (e.g., cooking skills, interpreting labeling, portion size, and ingredients lists) *“lack of knowledge is a limiting factor, so my options in terms of fiber foods are limited*”; (ii) convenience (e.g., accessibility and time) *“I try and spend the least amount of time cooking, so I have more time for other things*”; (iii) preferences (e.g., childhood exposure and variety) *“family eating key role in learning what food combinations work*”; (iv) cost (e.g., expensive and no deals) *“cheapest meals may not have a huge amount of fiber*”; (v) culture (e.g., eating out, trust, and cheap vs expensive) *“restaurants always give white rice*”; and (vi) side effects (e.g., heavy, stodgy, and bloating) *“fiber is associated with being a heavy type of food*”.

Two overriding themes emerged relating to dietary fiber-specific resources, namely, education to improve knowledge (e.g., healthy eating in schools, community focus, supermarket involvement, and cooking classes) *“schools have a key role in promoting healthy eating*” and public appeal (e.g., trusted sources/information, similar messaging to 5-a-day, advertising, and initiate conversation) *“consistency in information – changes over the years*” (Figure 3 and Table 2). Consumers were also keen to learn more relating to three key areas: (i) examples of dietary fiber-rich foods *“a long list of high fiber stuff*”; (ii) role of cooking *“more information on cooking and how this impacts fiber content – which method is better? (e.g., raw, boiling, or steaming)*” and making meals *“ready steady cook style*”; and (iii) labeling *“hard to visualize the portion size without scales*” and *“user-friendly ingredient lists*” (Figure 3).

### Staple foods and bread types

Overall, consumers were positive *per se* to the idea of dietary fiber-rich staple foods *“if more fiber in foods general probably will not be a bad thing*”; however, with various caveats *“keep same taste/look, nothing artificial and fiber without realising*” (Table 2). For example, the quality (e.g., shelf-life) taste, and cost must be maintained *“if it tasted the same and no change in cost*” as well as suggestions of the introduction of such foods at an early age *“if children grow up with high fiber foods they would get used to it*” and try before you buy *“in theory it would be great, but I would need to try it to see*”. Consumers’ expectations relating to dietary fiber-rich bread were predominately sensory related: (i) appearance (e.g., brown color) *“fiber is brown*”; (ii) taste *“like normal bread – exactly the same – otherwise no one will be eating it*”; and (iii) texture *“seeded*”.

**FIGURE 4:**
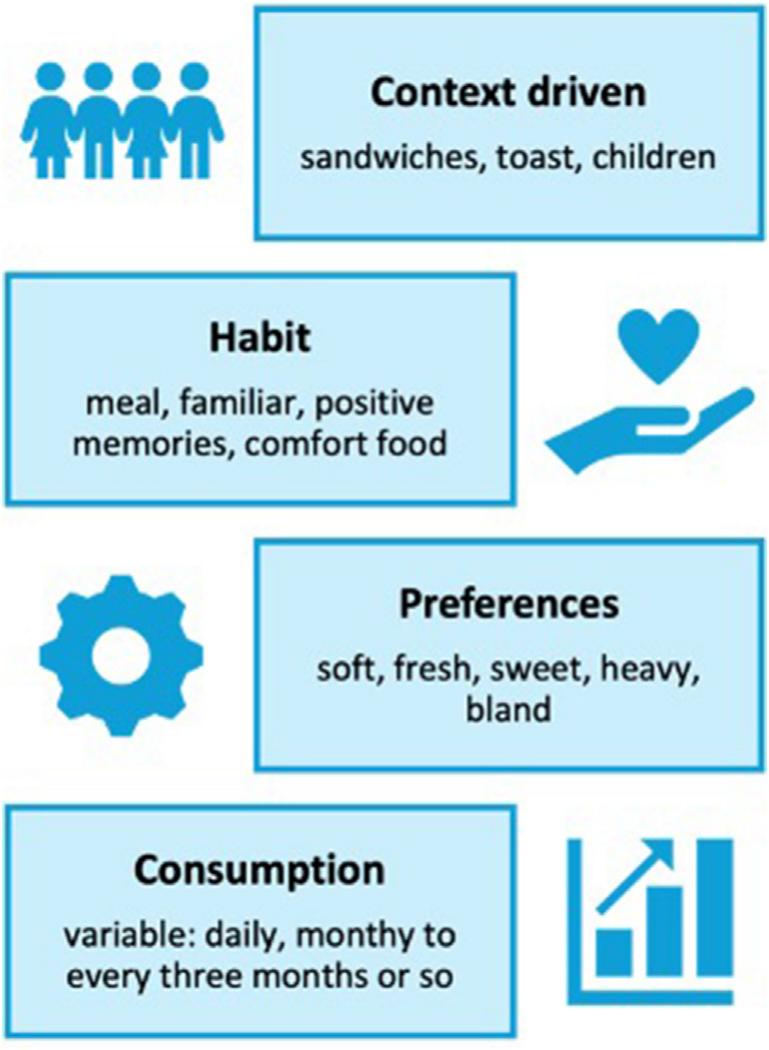
Overview of consumers’ key white bread-related trends.

Consumers mainly consumed white, wholemeal/brown, and seeded bread. In addition, other bread types (e.g., sourdough, granary bread, 50:50, and baguette) were consumed but to a less frequent extent. Bread’s functional role in the diet was also noted: *“from a loaf of bread, I know how many sandwiches I can make*.” More specifically, key trends relating to white bread were centered on three areas: (*1*) context is driving consumption such as *“white bread toasts really well*” and *“I associate sandwiches with white bread*”; (*2*) habit from positive memories *“comforting – it is what you are used to*” and meal *“I have a meal if white bread is in the house*”; and (*3*) preferences *“white bread must be soft and fresh*” (Figure 4). In addition, white bread consumption was notably variable from daily to less often *“some weeks loads and other less*” (Figure 4 and Table 2). Consumers noted that their preferred labeling strategy for dietary fiber-rich white bread was predominately focused on transparency *“explain things to us*”, awareness *“needs to be visible without looking back-of-pack with a magnifying glass*” and health conditions *“it is worrying if I have more fiber without being told it could upset my* *diet*”.

### Bread tasting

Consumers provided a range of comments relating to the breads (Table 3). Overall, it was clear that the control was considered to be like a standard white bread, off-white, fresh, sweet/artificial, and soft/sticky *“this is more like it – I knew it*” and *“standard white loaf*.” The Minax breads were characterized as follows: (*1*) Minax-100 was considered the most different bread as it looked different/less attractive, color/aroma differences, salty taste, and textural changes (e.g., stodgy, heavier, chewy, body, and bubbles) *“stodgy and more chewy*” and (*2*) Minax-168 was perceived to taste like bread, whiter in color, sour aroma, salty/sour taste, and springy *“very white like it* *is been bleached*”. In total, 45% of consumers perceived Minax-168 as the most preferred bread closely followed by the control (36%) and the least preferred bread was Minax-100 (19%). There was a mixed consensus in terms of purchase intention for the bread such as positively *“if nutritious and high in fiber or in meal deal may consider it*” and *“if money was no object I would buy A [Minax-168]*” vs negatively *“lots of persuasion to switch*” and *“I won’t buy C [Minax-100] as doesn’t look right*.” Consumers suggested changes for the bread where in most cases these were predominately for Minax-100 and texture-based (e.g., less chewy, dry, and pasty/sticky) *“is dry, scaly and different*”. In addition, comments related to modulating Minax-100 and Minax-168 aroma *“smelt weird to me – not exactly what it was and different to normal*”. Overall, it was apparent some consumers struggled to articulate how to improve the breads *“can’t really say without butter*”. Most consumers perceived Minax-100 as higher in dietary fiber due to textural changes *“more body*” and color differences *“we are all sitting here thinking fiber is brown*”. Consumers were also asked if they would modulate their future dietary fiber intake and this resulted in two themes: (*1*) initiated conversation/educational such as saying the session was beneficial *“learning lots today*” and *“I will go home and look up what fiber does*” and (*2*) highlighted positive intentions yet challenging to implement *“maybe for a bit – unlikely to maintain*”.

**TABLE 3:** Summary of consumers' key bread-tasting comments and preference

Themes	Control	Minax-100	Minax-168
Overall	Standard white bread, nice, crust	Looks different/less attractive	Tastes like bread
Appearance	Off-white	Color difference	Whiter
Aroma	Fresh	Smells different (sour)	Sour
Taste + flavor	Sweet, artificial	Salty, bland	Salty, sour, not sweet
Mouthfeel	Soft, sticky	Soft, stodgy/heavier, chewy, body, bubbles/scaly	Soft, springy
Preference^1^	15/42	8/42	19/42

1 Consumers (*n* = 42) were asked to select their most preferred bread.

## Discussion

### Food choice

It is important to understand the key factors in food choice to help ensure that nutritious, healthy, and sustainable foods are readily available for all. As expected in the current economic climate (e.g., cost-of-living crisis), cost was a driver for food-based decisions in most cases, coupled with convenience (accessibility, easy to cook, and shelf-life), nutrition/health aspects, sensory appeal (appearance, taste, and flavor), and trust (brands, packaging, family, and friends). Such findings also reflect the key factors such as food (e.g., sensory, nutritional/health information, and social/physical environmental), individual differences (e.g., biological, physiological, and psychological), and societal (e.g., culture, economic, and political) evident in the literature [12]. Practically, this can result in challenges in finding the balance in terms of cost vs healthy foods and access to nearby supermarkets as well as the role of food-related trust in food choice; similar findings were demonstrated from community-based interviews conducted in the North of England (Liverpool) [39]. In addition, a recent review highlighted that materials (e.g., local food environment, money, housing, and transport), meanings (e.g., food for all, autonomy, independence, community, health, and freshness), and competencies (e.g., poor mental and physical health, intake vs expenditure, and learning) were dominating themes in disadvantaged communities from a qualitative food perspective [40]. More broadly, it is vital that any new product (e.g., white bread higher in dietary fiber) avoids such pitfalls. For example, it is apparent that a new white bread higher in dietary fiber needs to deliver on being cost-effective, accessible from main supermarkets, nutritious, clearly labeled (source of fiber or high in fiber), and tasty so as to ensure uptake and suitability for the target market.

### Dietary fiber knowledge and awareness

Consumers need to have sufficient knowledge and awareness to make informed diet-related decisions. It was clear that dietary fiber was associated with confusion and poor awareness in most cases. Four key dietary fiber knowledge-related themes emerged: (*1*) lack of clarity relating to benefits (such as strong link with digestive function but unaware of disease risk aspects); (*2*) uncertainty of dietary fiber-rich sources and the role of marketing increasing awareness for certain foods (e.g., breakfast cereals); (*3*) misinterpretation of dietary recommendations (value related and confusion with 5-a-day); and (*4*) poor accessibility for dietary fiber labeling (e.g., back-of-pack and small font size). Interestingly, previous focus group-based studies have also highlighted the lack of knowledge relating to dietary fiber (e.g., benefits, recommendations, and identification) as noteworthy challenges to consumption [9,25,30]. Such findings are likely to explain the low dietary fiber consumption evident in the UK and globally [2,41,42]. Overall, this suggests dietary fiber is not at the forefront of consumers’ minds subsequently contributing to the low knowledge and awareness; accordingly, emphasis should be placed on consumer-centric approaches to promote uptake.

### Dietary fiber consumption and engagement

Capturing consumers’ current consumption and engagement habits can help in identifying any relevant areas for future focus. It was evident that consumers were not measuring their food intake and focused on eating by feeling via familiar dietary fiber-rich foods (e.g., baked beans, breakfast cereals, fruits, vegetables, and brown rice/pasta) subsequently contributing to confusion in terms of meeting dietary fiber recommendations. More broadly, this suggests consumers have some awareness of the key dietary components (e.g., 49% of consumers eat healthily most of the time) yet measuring food intake from both a consumers’ and researchers’ perspective is not without substantive challenges [43,44]. Accordingly, developing a simple, quick, and valid method to measure dietary fiber intake in different populations as well as provide personalized advice, especially in a digital format is much needed.

Moreover, six dietary fiber-driven key barriers were identified (such as insufficient knowledge, convenience, individual preferences, cost, culture, and side effects) and are all likely to add to dietary fiber-consumption-related challenges; accordingly, it is fundamental that such barriers are overcome to increase dietary fiber intake. Similarly, lack of knowledge contributing to identification and meal incorporation issues as well as preferences over various sensory properties (e.g., taste and texture) have also been cited as key consumption barriers [9,25,30]. In addition, ensuring standardization of labeling and definitions is fundamental to help guide consumers appropriately [9,30]. This is especially relevant for the UK consumers as dietary fiber is usually reported on the back-of-pack (unless demonstrating a nutritional claim such as a source of fiber or high fiber); therefore, it is reliant on consumers having sufficient awareness to find such information [3,4,10]. Consumers also cited the cost implications of dietary fiber-rich foods and limited offers/deals; accordingly, it is likely that budget-related advice will resonate with consumers. Moreover, dietary fiber is associated with satiety effects; therefore, in the cost-of-living crisis, this could be increasingly relevant to help manage hunger if budgets are limited [43,45]. In addition, it should be noted that Scarborough et al. [46] modeled various scenarios using UK dietary recommendations and found adherence would not result in significant cost changes.

Positively, consumers would like education to enhance knowledge in different settings (such as schools, community, and supermarkets) and public health campaigns (e.g., similar to 5-a-day as easy to remember) from trusted sources on key topics namely examples of dietary fiber-rich foods, role in cooking, on nutritional content/meal preparation and understanding food labeling. Previously, dietary fiber-specific educational materials were perceived as helpful and well received in terms of learning something new, changing future dietary fiber intake, format liking, engaging content, and sharing with others in an aging population [11]. Therefore, expanding this approach at a population level could be beneficial as well as a cost-effective solution to help overcome the associated dietary fiber knowledge gap. In addition, improving accessibility such as more dietary fiber-rich products across different categories that are easily identifiable without changes in sensory appeal and cost would help to increase consumer awareness and promote uptake. Overall, this suggests combining education with a personalized touch (e.g., catering for individual preferences and how to make a meal from affordable ingredients already in the household in a “ready steady cook” style) could help to make it easier for naïve consumers to consume a dietary fiber-rich diet.

### Staple foods and bread types

Staple foods (e.g., bread, pasta, and rice) provide an ideal vehicle for fortification and are typically consumed daily to varying extents; therefore, enabling benefits at an individual and population level. Overall, consumers’ initial thoughts were positive relating to dietary fiber-rich staple foods, but they also had a few concerns relating to cost, taste, and quality. This suggests food neophobia could play a key role in the perception of new foods [32]. Moreover, giving consumers opportunity to “try before you buy” (e.g., via tasting pods in supermarkets) could be a solution to encourage uptake, and address any potential food neophobia concerns, without consumers worrying about the cost implications of buying a product. It was clear that consumers’ expectations toward dietary fiber-rich bread were sensory driven such as brown in color and no distinct taste; interestingly, there was a strong association with dietary fiber being brown. This misconception may relate to the growing debate of white vs brown rice/pasta/bread as well as the lack of awareness that dietary fiber is present in a wide range of food categories (such as fruits, vegetables, breakfast cereals, whole grains, nuts, seeds, peas, and beans) [10,47]. It was also important to check consumers’ current consumption habits where their main bread types were white, wholemeal/brown, and seeded bread. This aligns with current market research demonstrating that white and wholemeal/granary breads are most commonly consumed weekly in UK households [15]. More specifically, the consumers noted white bread was used for sandwiches (especially for children) and/or toast, considered a comfort food and needs to be soft/fresh with consumption very variable from daily to less often. In addition, consumers were asked about preferred labeling strategies for dietary fiber-rich white bread and transparency/visibility dominated the conversation. As alluded to earlier, this suggests that improved labeling by the government and/or food manufacturers such as adding dietary fiber to the traffic lights scheme on front-of-pack could help to bring dietary fiber to the forefront of consumers’ minds. This insight is valuable as ensuring dietary fiber-rich white bread delivers on such components will encourage consumers to make the switch. There is widespread potential for this approach because white bread is the market leader in terms of bread sales in the UK [14,15].

### Bread tasting

Finally, consumers tasted higher in dietary fiber white bread prototypes to gain initial feedback as well as help to overcome any potential concerns consumers might have relating to this concept. Positively, Minax-168 was the consumers’ most preferred bread which demonstrates the potential of our prototypes to fit into the white bread market. However, additional quantitative consumer insights (e.g., hedonic, acceptability and willingness to buy data, in-store supermarket trials, etc.) are warranted post further product development. More broadly, it was clear that consumers were able to notice the subtle differences between the three breads. For example, the dietary fiber-rich breads were characterized by visual, aroma, and textural changes in most cases; accordingly, such breads will now be subject to various recipe improvements to address the cited issues. Overall, this supports the sensory profiling results to some extent which highlighted appearance modifications (e.g., color differences) [21]. Going forwards, it is important that white bread is evaluated how it is commonly consumed (e.g., sandwich and toast forms) to ensure the prototypes match consumers’ needs.

Importantly, the focus groups were conducted in two different locations in Reading including in an area of deprivation (Whitley) [48]. Therefore, future research should include focus groups in different parts of the UK to overcome any potential regional differences as well as include all stages of the lifecourse (e.g., from children to older adults). In addition, capturing socioeconomic status information is also relevant to dietary fiber intake and white bread consumption; however, obtaining this data may result in some consumers not wishing to take part so a balance is needed to reach such communities.

### Conclusion

This study conducted focus groups capturing initial background on dietary fiber to tasting white bread varying in dietary fiber content. Positively, this approach resonated with consumers subsequently enabling seven insightful sessions, and the overall experience was considered educational in most cases. Overall, it was apparent that dietary fiber is not at the forefront of consumers’ minds and dominant themes emerged in terms of knowledge (benefits, foods, recommendations, and labeling), consumption (not measuring intake), barriers (convenience and knowledge), resources (education and public appeal), and topics (food examples and cooking). In addition, there was a positive reaction to staple foods being higher in dietary fiber; however, there was an expectation of no changes in terms of appearance, taste, and cost. Consumers’ main bread types (e.g., white, wholemeal/brown, and seeded breads) were as expected. More specifically, consumers noted that white bread is context-driven (such as sandwich and toast), considered comfort food, needs to be soft/fresh and consumption is fairly variable (daily to less often) as well as needs to be delivered on transparent/visible labeling for new dietary fiber-rich white breads. Overall, the newly developed breads were well received and Minax-168 was the most preferred by the consumers; thus, highlighting the potential of the initial prototypes. Moreover, a try-before-you-buy scheme may help with enticing more skeptical consumers to make the switch as well as ensuring that the bread is delivered on being cost-effective, accessible from main supermarkets, nutritious, and clearly labeled. Accordingly, this suggests there is a need to help consumers increase their dietary fiber-related knowledge via education (e.g., food-based examples and role of cooking and labeling) and a personalized element, which could lead to noteworthy public health implications. In addition, a collective effort from the government and food industry as well as the consumer is necessary to ensure a step-change in dietary fiber consumption at an individual and population level.

## Acknowledgments

We would like to thank Dr Trisha Bennett and Emmaleigh Williams for their help in organizing the focus group sessions at Whitley Community Centre.

## Author contributions

The authors’ contributions were as follows – VN, CW, JRG, AL, PS, MC, NG, MJT, SL: designed research; VN, SL: conducted research; VN, SL: analyzed data; VN: wrote the original draft; VN, CW, JRG, AL, PS, MC, NG, MJT, SL: reviewed and edited the paper; SL: had primary responsibility for final content; and all authors: read and approved the final manuscript.

## Conflict of interest

The authors report no conflicts of interest.

## Funding

This research was funded by a UK Research and Innovation (UKRI) Biotechnology and Biological Sciences Research Council (BBSRC) grant (Hi-Fi Bread: Increasing UK Dietary Fiber – The Case for the Great White British Loaf – BB/W01792X/1).

## Data availability

Data described in the manuscript and code book will be made available upon request.
