# Supplementary material for: “Wait, Do I Need More Fiber?” Exploring UK Consumers’ Dietary Fiber-Related Awareness and White Bread as a Viable Solution to Promote Subsequent Intake
Source: Curr Dev Nutr. 2024 Jul 26;8(9):104430. doi: 10.1016/j.cdnut.2024.104430 (PMC11401220; doi:10.1016/j.cdnut.2024.104430)
Supplement: multimedia component 1 [file mmc1.pdf]

“WAIT, Do I Need More Fiber?” Exploring UK consumers’ Dietary Fiber-Related Awareness and White Bread as a Viable Solution to Promote Subsequent Intake

SUPPLEMENTARY

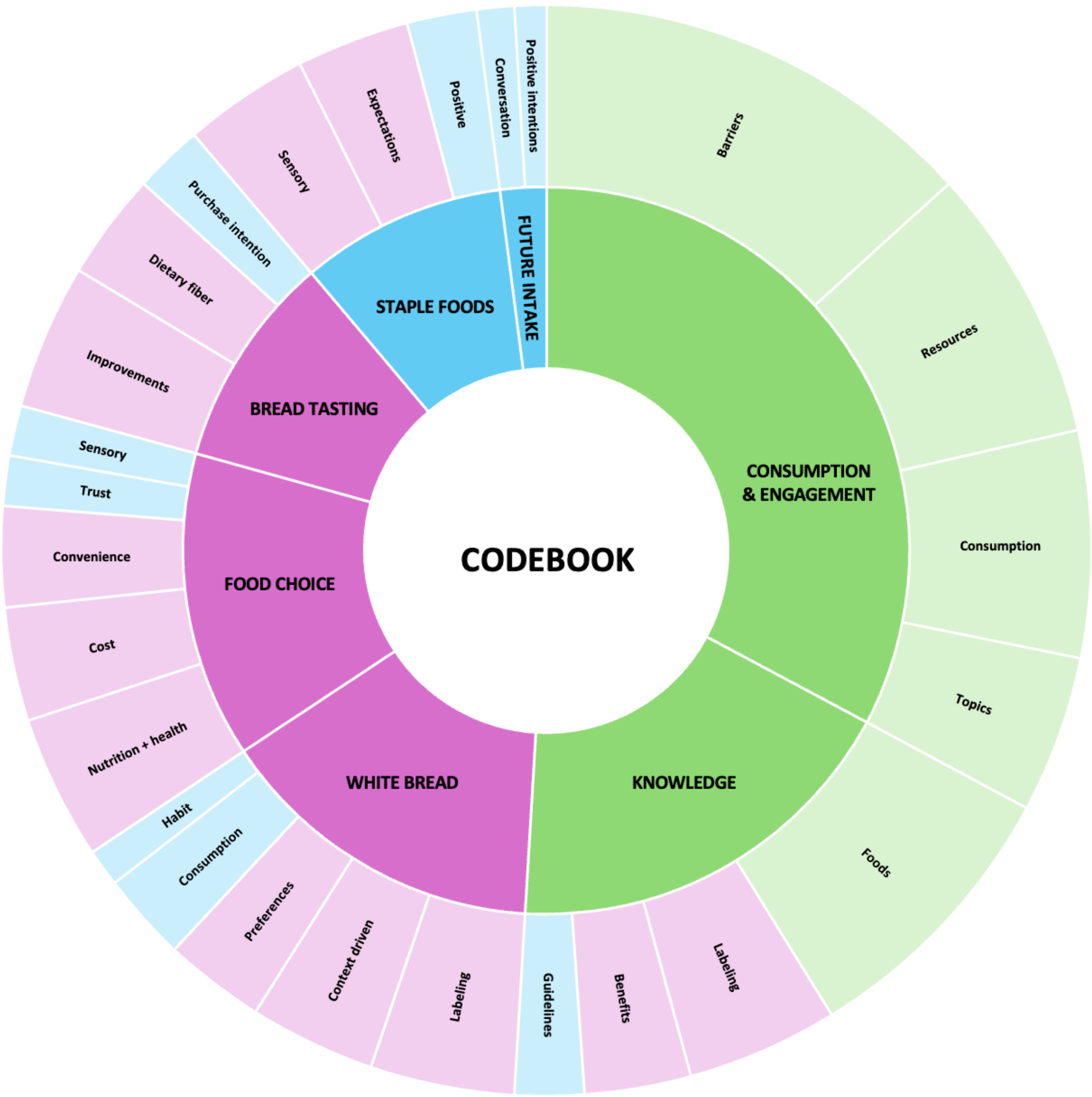

Figure S1. Codebook summary by overriding themes (high: more than 100 mentions (range: 120-188); medium: 50-99 mentions (range: 51-99); and low: less than 50 mentions (range: 11-43)) and subsequent codes (high: more than 25 mentions (range: 25-71); medium: 15-25 mentions (range: 16-24); and low: less than 15 mentions (range: 5-14) by frequency (e.g., number of times mentioned by consumers)).
